# Artificial intelligence for vascular minimally invasive surgery: techniques, clinical integration, and challenges

**DOI:** 10.3389/fcvm.2026.1966224

**Published:** 2026-10-22

**Authors:** Cenk Jiang, Bogdan Bortnovskii, Ijaz Gul, Mehmed Özkan, Lian Zhang, Dongmei Yu, Vijay Pandey, Runming Wang, Jian Wu, Peiwu Qin

**Affiliations:** 1 Institute of Biopharmaceutical and Health Engineering, Tsinghua Shenzhen International Graduate School, Shenzhen, China; 2 Chinese Medicine Guangdong Laboratory, Zhuhai, Guangdong, China; 3 Department of General Surgery, Hebei Key Laboratory of Colorectal Cancer Precision Diagnosis and Treatment, The First Hospital of Hebei Medical University, Shijiazhuang, China; 4 Institute of Biomedical Engineering, Boğaziçi University, Istanbul, Türkiye; 5 Medical Artificial Intelligence Lab, The First Hospital of Hebei Medical University, Shijiazhuang, China; 6 Department of Oncology, The First Hospital of Hebei Medical University, Shijiazhuang, China; 7 School of Mechanical, Electrical and Information Engineering, Shandong University, Weihai, China; 8 Translational Medicine Research Center, Chongqing University Three Gorges Hospital, Chongqing University, Chongqing, China

**Keywords:** artificial intelligence, ethics, medical robotics, minimally invasive surgeries, vascular intervention

## Abstract

**Introduction:**

Minimally invasive surgery (MIS), including endovascular and robot-assisted procedures, is widely used for vascular pathologies but remains limited by technical complexity, cognitive workload, and postoperative complications. Artificial Intelligence (AI) may help address these challenges through predictive analysis, decision support, image analysis, and automation.

**Methods:**

A structured Google Scholar search was conducted from February 21, 2025, to January 1, 2026, and updated through September 2026. Peer-reviewed studies applying AI to vascular MIS were included, with additional targeted searches addressing clinical translation, deployment, regulation, and ethical considerations.

**Results:**

Thirty primary studies were analyzed, including eight on machine learning, nine on deep learning, four on natural language processing, and nine on robotics or reinforcement learning. Machine learning supported risk stratification and outcome prediction, deep learning enabled imaging and segmentation applications, natural language processing facilitated clinical information extraction, and robotic systems supported navigation and procedural assistance.

**Discussion:**

AI demonstrates substantial potential across the vascular MIS pathway, but broader clinical adoption requires stronger validation, prospective multicenter studies, standardized reporting, health-economic evaluation, and careful consideration of regulatory, liability, and accessibility challenges.

## 1 Introduction

Vascular pathologies, often complicated by cardiac and non-cardiac comorbidities, pose a significant threat due to the rising prevalence of smoking, alcohol consumption, obesity, and diabetes among youth (1). Surgically treatable vascular conditions, including aneurysms, arterial dissections, embolism, and ischemia, may present with diverse clinical complications and hence require precise, personalized treatment to minimize perioperative (i.e., preoperative, intraoperative, and postoperative) risks (2). In particular, a comprehensive analysis by Fowkes et al. examined the global prevalence of peripheral artery disease (PAD), a vascular condition associated with impaired mobility, across high-, middle-, and low-income countries, using data obtained from 34 community-based studies with 112,027 participants (3). The results indicated that 202 million people were living with PAD in 2010, a 23.51% increase from 2000, driven by the population of low- and middle-income countries, which accounts for 69.7% of 2,010 global PAD cases. Moreover, a recent study by Zhou et al. (4) estimated that approximately 316.5 million people aged 25 yr and older were affected by PAD worldwide in 2023, corresponding to a global prevalence of 6.58%.

Importantly, PAD has been associated with a threefold increase in the risk of cardiovascular mortality, highlighting its substantial contribution to global cardiovascular mortality (3). Other vascular diseases similarly contribute to substantial morbidity and mortality, establishing the need for effective minimally invasive surgery (MIS) approaches. The introduction of MIS has, in turn, revolutionized vascular disease management by reducing the size of interventional incisions (5–7). This facilitates reductions in procedural trauma, recovery time, pain, and mortality rates, especially among vulnerable patient groups (8, 9).

Minimally invasive procedures typically include endovascular interventions, hybrid approaches, and robot-assisted surgeries (10). Specifically, endovascular interventions are widely recognized as the first-line treatment options for several vascular conditions, including peripheral arterial, carotid, and venous diseases, as well as aneurysms (11–14). Collectively, these vascular disorders contribute substantially to global morbidity and mortality. Endovascular surgeries are performed with atraumatic devices, such as catheters, soft-tip wires, balloons, expandable stents, and grafts, introduced either through cut-down or percutaneous access (15). Similarly, hybrid procedures are preferred in more complicated conditions, such as the treatment of aortic arch and thoracoabdominal aortic pathologies, as well as coronary revascularization (16, 17). This permits a greater extent of treatment personalization, which is important for high-risk patients (16).

Robotic technology has also been applied to endovascular systems, enabling remote catheter manipulation, guidewire navigation, and device delivery during complex vascular interventions. Furthermore, these systems can provide independent torque control and active maneuverability of both the catheter and the wire, while enhancing intravascular navigation and device placement (10, 18). More recently, magnetically guided tethered probes (i.e., partially submersible) and untethered microrobotic devices (i.e., completely submersible) have also been investigated for intravascular navigation and therapeutic delivery (19).

Each of the minimally invasive approaches discussed above offers the advantages of reduced surgical footprint and an expanded toolkit for the more challenging clinical cases (20). These advantages can mitigate the risks of surgical complications, shorten intensive care unit stays, facilitate recovery, and lower the mortality rate (21). In addition, certain endovascular procedures, such as elective endovascular abdominal aortic aneurysm repair, can be performed under local anesthesia in selected patients, enabling treatment for those who are ineligible for general anesthesia and broadening access to medical treatment for a wider population (22). However, the high cognitive workload demanded by complex MIS, the lack of large-scale randomized clinical trials, and the high costs of developing and implementing minimally invasive surgical strategies in specialized facilities hinder their widespread adoption (23, 24). A higher rate of technical failures, compared to open surgeries, increases the risks of endoleaks, requiring reintervention, potentially necessitating more radical solutions, such as amputations (25, 26).

As these challenges persist in MIS, the adoption of AI has grown rapidly across healthcare, offering advanced technologies and innovative solutions to improve patient outcomes. This growing trend is also evident in the increasing number of publications investigating machine-learning techniques across vascular surgery between 1991 and 2021, particularly for diagnostic, prognostic, and image-segmentation tasks (27).

The integration of AI has been focused on the fields of medical image screening and diagnostics, patient monitoring, robotics, medical research, and drug discovery (28–31). In medical image screening, advanced deep learning (DL) algorithms have been used for real-time dataset analysis, image segmentation, and object detection for various tumors, improving operational efficiency (32). In patient monitoring, machine learning (ML) algorithms have been applied to electronic health records of intensive care unit patients for early mortality detection (33), potentially supporting patient risk stratification. Similarly, DL models have been applied to electrocardiographic data for stroke risk prediction, highlighting the value of AI integration in healthcare (34). In drug discovery and research, metabolomics combined with machine learning techniques has been used to identify plasma metabolites that serve as early biomarkers for lung cancer (35). Among these fields, endovascular surgeons could benefit from AI adoption in perioperative patient monitoring, including ML-based risk stratification and DL-based imaging models for preoperative planning, voice-controlled virtual assistance systems integrated with large language models (LLMs), and real-time image segmentation models for intraoperative assistance, as well as prognostic tools to guide postoperative care (Figure 1).

**Figure 1:**
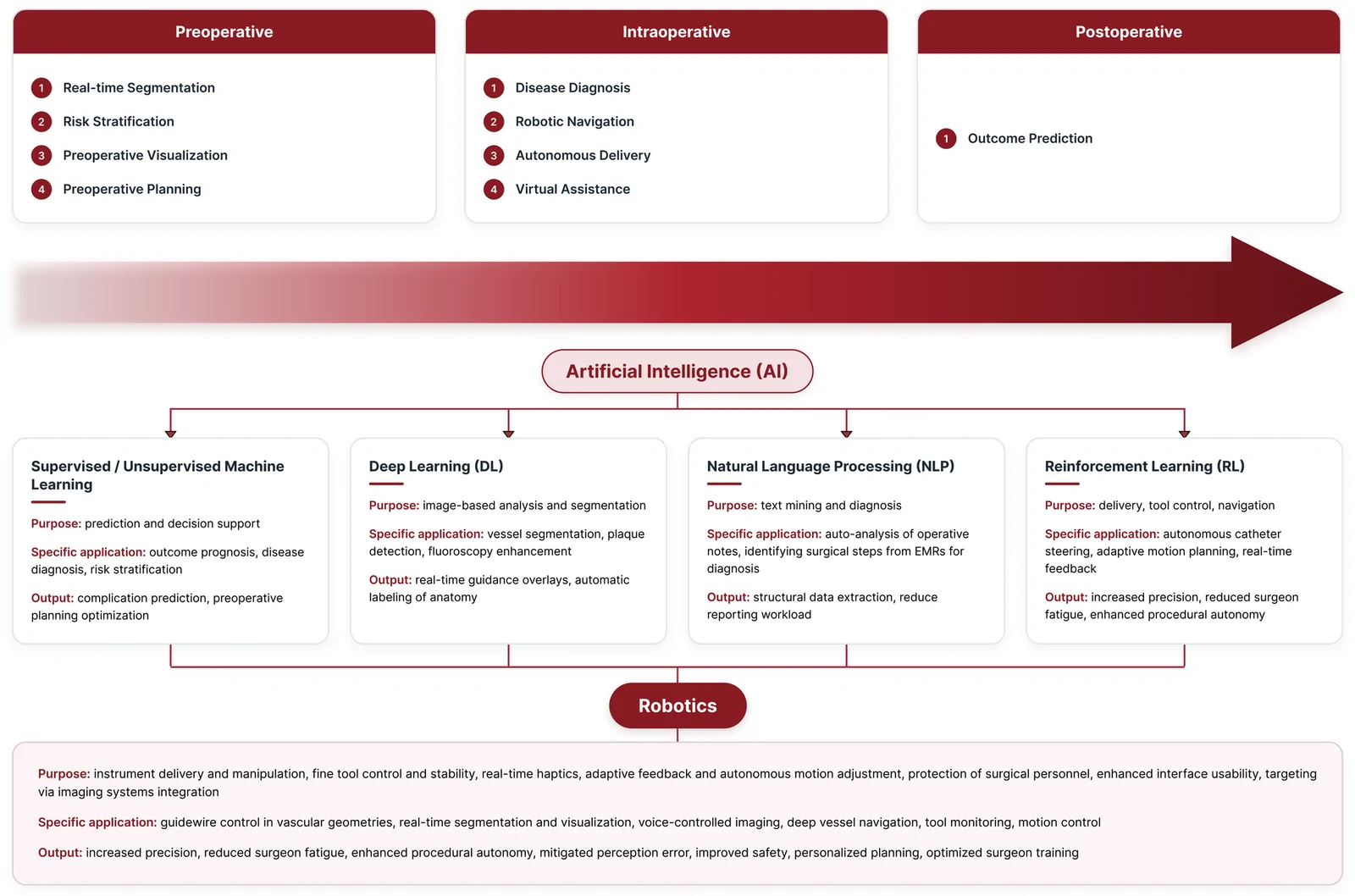
Overview of AI applications and enabling techniques across the perioperative workflow in vascular MIS. The upper panel depicts AI-supported tasks across the preoperative, intraoperative, and postoperative stages, including diagnosis, risk stratification, visualization, procedural planning, robotic navigation, autonomous delivery, virtual assistance, and outcome prediction. The lower panel links these clinical functions to their underlying AI paradigms, highlighting their respective purposes, representative applications, and clinical outputs.

It is evident that AI is rapidly advancing in healthcare. This advancement has also transformed endovascular surgeries, resulting in state-of-the-art innovations that need further exploration. This review aims to provide a comprehensive overview of recent AI techniques applied in endovascular surgeries, analyze the translational pathway and clinical deployment of AI-based systems in minimally invasive surgery, identify current methodological and practical limitations, and outline future perspectives for the development and adoption of AI in this field. In the following section, four key AI techniques are reviewed: ML, DL, Natural Language Processing (NLP), and Robotics with Reinforcement Learning (RL).

## 2 Methodology

This narrative review aimed to examine current AI applications in vascular MIS, assess the translational pathway, and identify the principal barriers to clinical implementation.

A structured literature search was conducted using Google Scholar between February 21st, 2025, and January 1st, 2026, and was updated during manuscript revision through September 2026. A wide range of keywords was used, including “artificial intelligence,” “machine learning,” “deep learning,” “natural language processing,” and “robotics,” each followed by “vascular minimally invasive surgery” or “endovascular intervention.” Targeted searches were additionally performed to identify literature regarding the translational pathway, clinical deployment, regulatory frameworks, and ethical considerations.

Inclusion criteria were (1) peer-reviewed publications that employed artificial intelligence as the primary method in vascular MIS and (2) peer-reviewed publications that focused on clinical translation, regulatory frameworks, ethical considerations, or implementation of AI in vascular or endovascular MIS. Exclusion criteria were (1) publications that did not include artificial intelligence, (2) publications that focused on interventions unrelated to vascular pathologies, (3) publications that were inaccessible as full text, and (4) abstract-only publications, preprints, unpublished manuscripts, and non-peer-reviewed material.

Following the search and screening process, all eligible articles were reviewed in detail and classified according to their respective AI categories, including machine learning, deep learning, natural language processing, and robotics with reinforcement learning (Figure 2).

**Figure 2:**
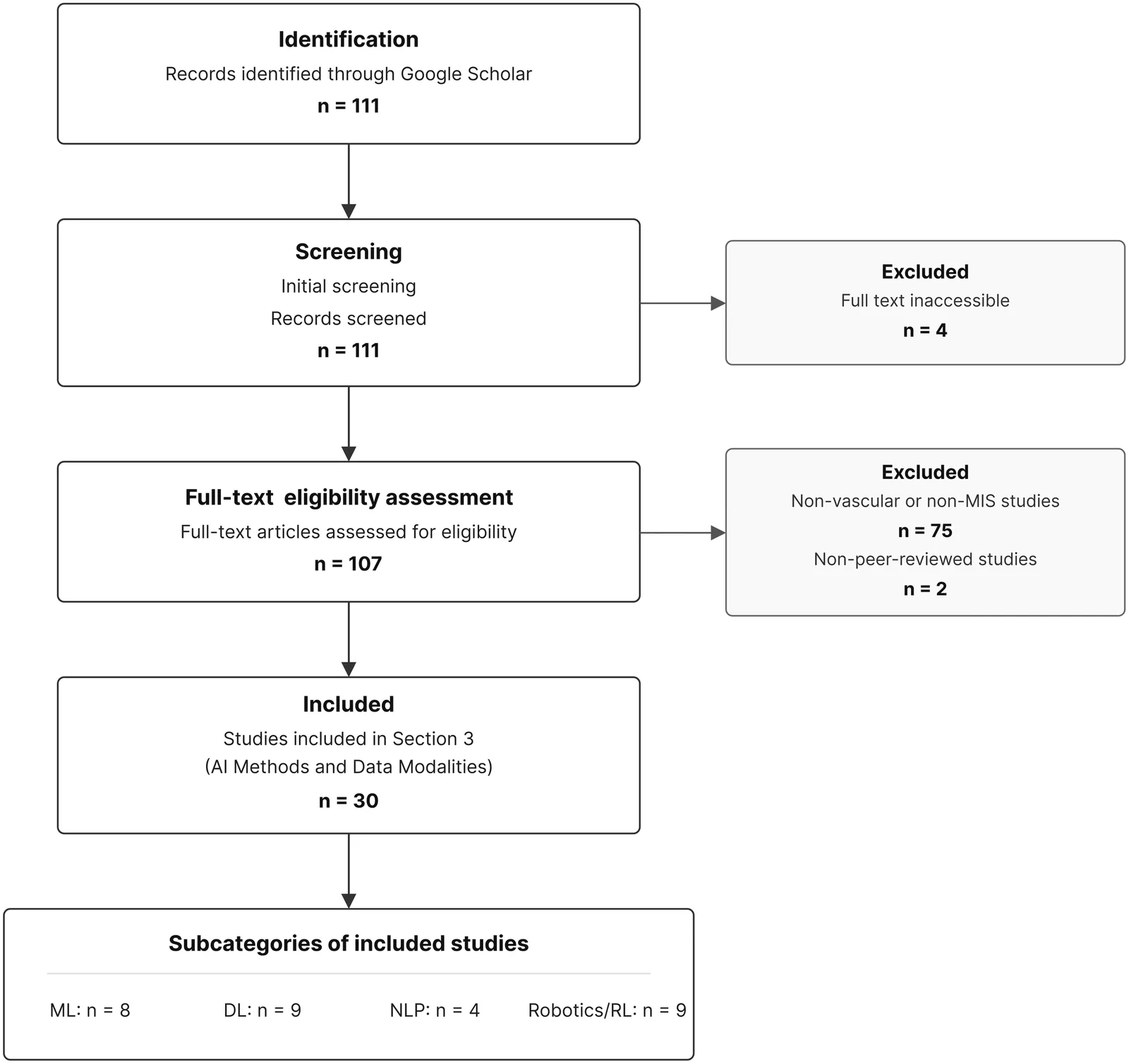
The study-selection flowchart summarizes the identification and screening of primary research studies included in Section 3 (AI Methods and Data Modalities). Literature used to support the Introduction, Translational Pathway and Deployment, Challenges and Ethical Considerations, and Future Perspectives sections was identified through separate targeted searches and was not included in this flowchart.

## 3 AI methods and data modalities

### 3.1 Machine learning

ML, at its core, is a subfield of AI that enables computers to make predictions. Training an ML algorithm generally involves a three-stage workflow: the forward pass, which extracts key features and computes predictions through input data; backpropagation, which calculates the error between predicted and target outputs; and gradient descent, an optimization algorithm that adjusts the parameters to minimize the error (Figure 3). Once trained, an ML algorithm utilizes an optimized computational model to map inputs to predicted outputs, making it a highly versatile and widely adopted technique across multiple domains (36). Broadly, ML can be categorized into two main subtypes: supervised and unsupervised learning. Supervised ML requires labeled outputs during the training phase, whereas unsupervised ML does not require labeled outputs to conclude an input-output mapping (37). The ML studies discussed in this subsection are summarized in Table 1.

**Figure 3:**
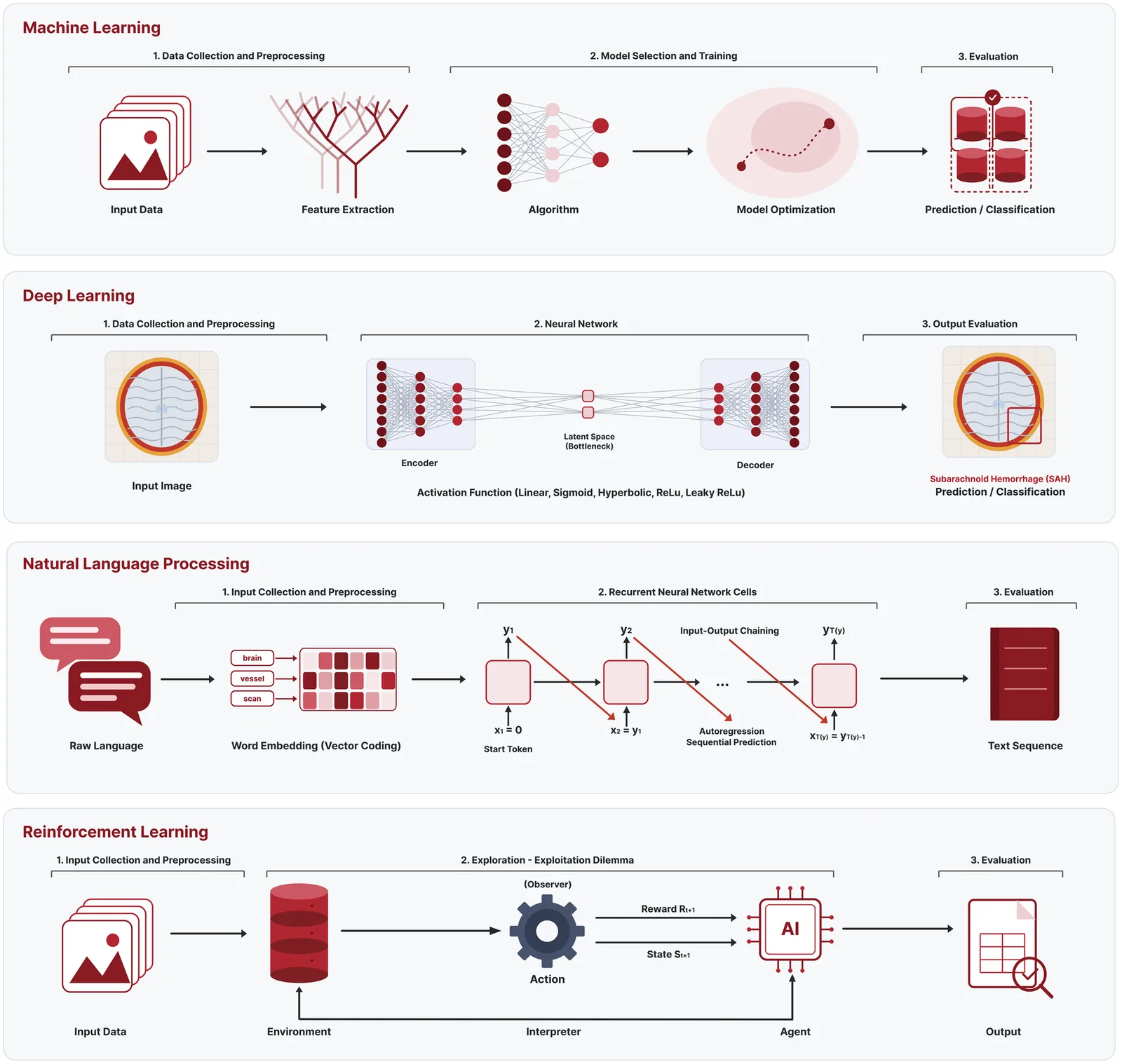
Architecture of four key AI techniques used in endovascular surgery: machine learning, deep learning, natural language processing, and reinforcement learning.

**Table 1:** AI in endovascular minimally invasive surgery: machine learning.

Study	Sample data	Prevalence	Reported results	Val. and Cal.
Li et al. (38)	VQI: 235,677 patients	30.4%	AUROC: 0.94; Acc: 0.86	Internal, Brier: 0.09 (pre), 0.08 (intra), 0.06 (post)
Li et al. (39)	VQI: 38,325 patients	7.0%	Postoperative AUROC: 0.94; Acc: 0.87	Internal, Brier: 0.10 (pre), 0.09 (intra), 0.08 (post)
Li et al. (40)	VQI: 33,924 patients	16.6%	AUROC: 0.94; Acc: 0.85	Internal, Brier: 0.12 (pre), 0.11 (intra), 0.10 (post)
Li et al. (41)	ACS NSQIP: 6,601 patients	7.1%	AUROC: 0.94; Acc: 0.86	Internal, Brier: 0.08
Ross et al. (42)	GenePAD study: 1,755 patients (1,047 used for model analysis)	PAD: 183/1047, mortality: 129/1047	AUROC: 0.87 (identification); 0.76 (prediction)	Internal, Hosmer-Lemeshow P-value (best models): 0.70 (PAD), 0.62 (mortality), 0.06 (undiagnosed-PAD)
Huang et al. (43)	PERSIST: 525 patients	54.1%	AUROC: 0.72 (internal); 0.68 (external)	External, MCE: 0.088 (internal); 0.091 (external)
Nishibe et al. (44)	Collected data: 169 patients	NR	Acc: 68.7%; Sens: 79.0%; Spec: 65.7%	Internal, NR
Guo et al. (45)	Collected data: 10 surgeons	N/A	Acc: 0.97 (with VD); 0.87 (without VD)	Internal, NR

ACS NSQIP, American College of Surgeons National Surgical Quality Improvement Program; AI, artificial intelligence; Acc, accuracy; AUROC, area under the receiver operating characteristic curve; Cal., calibration; GenePAD, Genetic Determinants of Peripheral Arterial Disease; MCE, maximum calibration error; N/A, not applicable; NR, not reported; PAD, peripheral artery disease; PERSIST, Posterior Circulation Ischemic Stroke Registry; Sens, sensitivity; Spec, specificity; VQI, Vascular Quality Initiative; VD, vascular difficulty; Val., validation; pre, preoperative; intra, intraoperative; post, postoperative.

ML algorithms have emerged as widely adopted prognostic tools in endovascular surgery, supporting postoperative care and outcome prediction. Several studies have evaluated the predictive performance of multiple ML models across different endovascular procedures, yielding valuable clinical insights.

A series of studies by Ben Li and colleagues assessed related ML frameworks across several vascular interventions. Using data from the Vascular Quality Initiative (VQI), Li et al. (38) developed multiple ML models to predict major adverse limb events or mortality one year following endovascular surgery for PAD. The findings demonstrated that the eXtreme Gradient Boosting algorithm (XGBoost), a scalable end-to-end tree-boosting framework (46), achieved the strongest performance, with an area under the receiver operating characteristic curve (AUROC) of 0.94 and an accuracy of 0.86. Related VQI-based studies from the same research group deployed similar models to predict stroke or mortality and lack of clinical improvement after transcarotid artery revascularization (39), and endovenous vein ablation (40), respectively. Consistent with previous findings, XGBoost achieved the highest predictive performance across these applications, highlighting the practicality and potential utility of ML models for postoperative predictions. In a separate study by Ben Li and colleagues, the American College of Surgeons National Surgical Quality Improvement Program (ACS NSQIP) database was utilized. Li et al. (41) applied ML models to predict 30-day outcomes following endovascular aortoiliac revascularization for aortoiliac occlusive disease, a distinct subset of PAD. The model’s prediction capability was further extended to secondary outcomes, including re-intervention, amputation, major adverse cardiovascular events, bleeding, wound complications, readmission, and others, to assess performance across additional clinical outcomes. Consistent with earlier findings, XGBoost demonstrated superior predictive performance, achieving an AUROC of 0.94 with an accuracy of 0.86 for primary outcomes, and AUROC values ranging from 0.86 to 0.97 with accuracies between 0.78 and 0.93 for secondary outcomes, outperforming logistic regression and existing predictive tools.

Although these studies examined different procedures, outcomes, and patient cohorts, their findings should be interpreted as a related body of evidence rather than as independent confirmation of model generalizability. The first three studies were derived from the VQI database and were conducted by largely the same researchers using similar models, while the aortoiliac study used the ACS NSQIP database.

Beyond the XGBoost framework, several supervised ML algorithms have also been evaluated against logistic regression for both disease identification and outcome prediction. Ross et al. (42) assessed the performance of Elastic Net, a penalized regression model, and Random Forest classifiers for PAD identification and long-term mortality prediction. The results demonstrated that Elastic Net outperformed logistic regression for PAD identification, achieving an area under the curve (AUC) of 0.87, while Random Forest demonstrated superior performance for detecting undiagnosed PAD (AUC of 0.84) and predicting eight-year mortality (AUC of 0.76).

ML algorithms have also been employed as risk stratification tools by classifying patients according to their potential surgical risks and outcomes. Huang et al. (43) developed a robust logistic regression model for risk stratification in patients with acute vertebrobasilar artery occlusion. Random Forest analysis was used to identify the National Institutes of Health Stroke Scale, symptomatic intracranial hemorrhage, and estimated occlusion-to-groin puncture time as the most influential predictors in this patient cohort. The model was validated using both internal and external datasets and demonstrated adequate predictive performance in identifying high-risk subgroups, thereby highlighting the clinical value of ML-based risk stratification for preoperative planning.

Moreover, Nishibe et al. (44) utilized ML-based decision tree analysis (DTA) to predict three-year mortality following endovascular aortic repair (EVAR) in 169 patients with abdominal aortic aneurysms (AAA). Out of 23 variables, poor nutritional status, chronic kidney disease, chronic obstructive pulmonary disease, and advanced age were identified as major predictors. The DTA model achieved an accuracy of 68.7%, with a sensitivity of 79.0% and a specificity of 65.7%, highlighting the potential of ML-based models as a preoperative risk stratification tool.

In addition, ML algorithms have been applied to assess surgeons’ operational skills in vascular intervention procedures. Conventional evaluation approaches rely on both subjective and objective metrics, such as operative speed, motion smoothness, and precision. However, these metrics often disregard patient-specific vascular conditions, including vessel diameter, branch distance, inclination angle at the aortic arch, and the overall anatomical complexity of the vasculature. To address this limitation, Guo et al. (45) developed an ML-based framework for objective skill assessment that integrates a novel Vascular Difficulty (VD) index. Support Vector Machine and Random Forest models were trained using both anatomical features and operative motion data, combining traditional and vascular descriptors. By incorporating the VD index, the skill classification accuracy improved by 10%, demonstrating the importance of vasculature-aware evaluation metrics for more objective skill assessment in vascular intervention surgeries (Table 1).

Despite the promising performance reported across these studies, independent external validation remains limited. Among the eight ML studies reviewed in this subsection, only Huang et al. (43) evaluated the model using an independent external cohort, consisting of 237 patients from 16 European stroke centers. The remaining studies primarily relied on internal evaluation, including random training-test splits or cross-validation. Moreover, several models were developed using large multicenter registries, including VQI and ACS NSQIP. However, internal testing within these databases still does not establish clinical generalizability.

### 3.2 Deep learning

DL is a sub-branch of ML designed to imitate aspects of human cognition through multilayered artificial neural networks (ANNs) (47). Due to their resemblance to neuronal circuits, ANNs, a major class of DL architectures, can be classified as a Biomimetic Intelligence tool utilizing nature-inspired algorithms for the assessment of biological, clinical, and imaging data (48) (Figure 3).

Their application in healthcare is particularly attractive due to the enhanced capability of DL networks to identify and continuously learn complex and subtle patterns from large-scale datasets (49). Furthermore, the strong potential for edge computing, bedside computation, and data storage enables personalized analytics and accelerated knowledge generation, thereby facilitating patient treatment (50). Based on our literature assessment, the main areas of DL application include numerical data processing for predicting operative and treatment outcomes, real-time visual segmentation, preoperative imaging, and surgical planning. The DL studies discussed in this subsection are summarized in Table 2.

**Table 2:** AI in endovascular minimally invasive surgery: deep learning.

Study	Application	Sample data	Reported results	Validation
Li et al. (51)	U-Net-based semantic segmentation of “No-Go” landing zones for EVAR guidance	Collected data: 369 images from 110 patients/videos	Primary: IoU: 0.43; F1: 0.53. Secondary: Acc: 0.97; Sens: 0.51; Spec: 0.99	Internal
Gao et al. (52)	Vessel segmentation accuracy through DBCM-integrated FR-UNet backbone	DRIVE: 40 images; CHASE DB1: 28 images	(DRIVE): Acc: 0.96; F1: 0.82. (CHASE DB1): Acc: 0.96; F1: 0.80	Internal
Laddi et al. (53)	Real-time venous structure segmentation	Collected data: 720 NIR images from 72 subjects	Dice: 0.58; Acc: 0.97	Internal
Zhao et al. (54)	GAN-based real-time guidewire path planning for endovascular interventions	Collected data: 350,000 samples, 10 surgeons	Path-planning Acc: higher than baseline (46.2%–69.78%) and non-rigid registration (72.94%)	Internal
Kurmann et al. (55)	Instance segmentation of surgical instruments	2017 Endoscopic Vision robotic instrument-segmentation challenge: 10 videos	mIoU: types, 0.66; parts, 0.65; Sequences 9/10, 0.73. mAP: validation, 0.50; test, 0.48	Internal
Wang et al. (56)	Transfer learning for vasculature-aware planning	Collected data: 40 patients (DSA); in vivo (22 mice)	Human: AUROC: 0.97; Avg. Dice: 0.66. Mice: Acc: 0.88	Internal
Saitta et al. (57)	3D U-Net for thoracic aortic segmentation and thoracic EVAR planning	Collected data: 465 CT scans	Dice: 0.95	Internal
Smorenburg et al. (58)	Multitask CNN for intraoperative endoleak detection and localization during EVAR	Collected data: 660 DSA maps, 220 patients	AUROC: 0.85; F1: 0.78; Precision: 0.95; Recall: 0.73	Internal
Wang et al. (59)	Operator behavior recognition and skill evaluation in PCI through multimodal data fusion	Collected data: multimodal sensor data from 10 interventionists	Acc: 0.99	Internal

AI, artificial intelligence; Acc, accuracy; AUROC, area under the receiver operating characteristic curve; Avg., average; CHASE DB1, Child Heart and Health Study in England Database 1; CNN, convolutional neural network; CT, computed tomography; DBCM, dual-branch convolutional module; DRIVE, Digital Retinal Images for Vessel Extraction; DSA, digital subtraction angiography; EVAR, endovascular aneurysm repair; F1, F1 score; FR-UNet, full-resolution network; GAN, generative adversarial network; IoU, intersection over union; mAP, mean average precision; mIoU, mean intersection over union; NIR, near-infrared; PCI, percutaneous coronary intervention; Sens, sensitivity; Spec, specificity; 3D, three-dimensional.

CNNs are a major class of DL architecture underlying modern computer vision (CV) systems, enabling machines to recognize and interpret images and video data (47, 60). Within MIS, a substantial amount of CV research focuses on image segmentation, a data processing task that enables the precise partitioning of images into distinct, information-rich regions (61). One of the most widely adopted CNN architectures for biomedical image segmentation is U-Net, named after its characteristic U-shaped symmetric encoder–decoder structure. The encoder progressively reduces spatial resolution to capture high-level features, forming the contracting path (62), while the decoder restores spatial resolution through repeated upsampling, capturing global contextual semantics and forming the expansive path. A defining feature of U-Net is its skip connections (referred to as “crop and copy” in the original work), which concatenate encoder and decoder features to preserve fine-grained spatial information and mitigate feature loss (62).

Compared with classical optimization-based segmentation approaches such as graph-cut methods, U-Net demonstrates substantial performance advantages. Livne et al. (63) demonstrated that both full and reduced (half) U-Net models consistently outperformed graph-cut techniques across all evaluation metrics. Specifically, the full U-Net achieved a Dice coefficient of 0.891, a 95th percentile Hausdorff distance of 47.277 voxels, and an average Hausdorff distance of 0.342 voxels, indicating superior segmentation accuracy. Moreover, U-Net exhibited strong generalizability in intra-model evaluations using 3-Tesla time-of-flight angiography, highlighting its significance for biomedical image segmentation.

Given U-Net’s established performance in biomedical image segmentation, Li et al. (51) presented a “No-Go” model based on semantic (i.e., pixel-level categorization) U-Net segmentation of radiographic anatomy to identify proximal contact zones during endovascular aneurysm repair. The model classified each pixel according to the presence or absence of the “No-Go” zone and was evaluated using 10-fold cross-validation on 369 fluoroscopic images, achieving an IoU of 0.43±0.29 and an F1 score of 0.53±0.32, with a pixel-wise accuracy of 0.97±0.002. These findings demonstrate the feasibility of using semantic segmentation to identify suboptimal stent-graft landing zones.

Similarly, Gao et al. (52) proposed a dual-branch convolutional module (DBCM), a DL-based vessel segmentation framework designed to address challenges related to low contrast, fine-scale structures, and morphological complexity. The DBCM was integrated into FR-UNet, a modified full-resolution backbone combining standard and depth-wise separable convolutions to capture both global semantic and local structural features. The framework further incorporated coordinate attention mechanisms and residual connections to enhance feature representation and facilitate stable gradient flow while maintaining computational efficiency. The framework was evaluated using training and test splits of DRIVE and CHASE DB1 retinal vessel datasets. On DRIVE, the framework achieved an accuracy of 0.9653 and an F1 score of 0.8156. On CHASE DB1, the corresponding values were 0.9631 and 0.8031, respectively. These results demonstrated improvements over the FR-UNet backbone on both datasets.

Laddi et al. (53) further demonstrated that a manually trained, self-parametrized U-Net provided higher accuracy in real-time segmentation and visualization of upper and lower extremity veins compared to CNN feature-based learning models such as residual network and visual geometry group network. These findings emphasize the value of U-Net-based frameworks associated with repeated vascular interventions, including venipuncture and chronic venous disease treatment.

Path planning is a crucial component of both preoperative planning and intraoperative guidance in vascular interventions, enabling the generation of optimal procedural trajectories. Zhao et al. (54) introduced Surgical-GAN, a generative adversarial network-based model that combines CNNs and long short-term memory networks, for real-time guidewire path planning. The model extracts spatial and temporal features to predict the local future path of the guidewire. The framework was trained on 350,000 samples collected by 10 surgeons and evaluated in a vascular model, achieving higher path-planning accuracy than baseline (baseline accuracy: 46.2%–69.78%) and a non-rigid registration method (non-rigid accuracy: 72.94%).

Instance segmentation combines pixel-level classification with object detection, enabling both semantic categorization and precise localization of individual objects. This approach has shown strong potential in surgical instrument recognition, as demonstrated by Kurmann et al. (55). The proposed architecture employed a shared encoder, two segmentation decoders, and a classifier, first isolating surgical tools into individual pixel-wise instances before assigning them to specific categories. Unlike semantic alternatives, this method relies on small-scale differences between the instrument types, as well as pose estimation or instrument tracking, offering a more informative map with the same level of accuracy and precision.

DL models have also been developed to characterize vascular anatomy for diagnostic and surgical planning applications. VasNet, a vasculature-aware unsupervised transfer learning framework for automated personalized anatomical structure generation, was introduced by Wang et al. (56). The model enables pathophysiological recognition from limited fluorescence and angiography datasets and supports surgical planning and image reconstruction. VasNet employs domain-adversarial loss functions and multi-scale feature fusion to distinguish vascular structures from background noise, demonstrating significant improvements in vascular diagnostics, blood flow assessment, and complication prediction.

In addition, the quality of DL-generated outputs depends strongly on segmentation granularity, which ranges from coarse to fine (64). Selecting an appropriate granularity level is therefore essential for extracting relevant information while maintaining an optimal detail-to-noise ratio. For instance, enhanced data informativeness in DL-based CV algorithms is achieved through multi-scale hierarchical feature extraction, employing multi-branch dilated CNN layers with kernels of varying receptive field sizes, followed by feature concatenation and fusion (65).

Moreover, Saitta et al. (57) developed a fully automated CNN-based 3D U-Net pipeline to detect proximal landing zones and quantify geometric features for thoracic EVAR planning and endograft deployment. The CNN-based framework also quantified additional clinically relevant geometric parameters such as landing-zone diameter, angulation, tortuosity, and aortic arch curvature. The model was trained on 395 computed tomography (CT) scans and evaluated on 70 independent test scans, achieving a mean Dice score of 0.95, while accurately segmenting additional pathological cases of thoracic aortic aneurysm.

Besides preoperative imaging, DL models have also displayed potential for intraoperative image analysis during vascular MIS. Smorenburg et al. (58) developed an intraoperative multitask DL framework for AAA patients to automatically detect and localize endoleaks from DSA-derived perfusion maps acquired during EVAR. A two-dimensional CNN-based U-Net model was trained with multitask learning on 660 DSA-derived perfusion maps obtained from 220 patients, achieving an AUROC of 0.85 and an F1 of 0.78. By simultaneously classifying and localizing endoleaks, this study demonstrates the potential of DL-based intraoperative image analysis.

Finally, DL-based multimodal fusion has emerged as a powerful strategy for integrating complementary information from heterogeneous data sources, leading to improved performance in operational skill assessment compared with traditional ML approaches. Wang et al. (59) introduced a DL-based data fusion network (DF-Net), a two-stream one-dimensional CNN architecture designed to recognize common operating behaviors of vascular interventionists by using physiological and motion sensor data. Sensor arrays placed on operators’ arms collected two data streams: electromagnetic tracking, inertial measurement unit, and pressure sensor data in the first stream, and surface electromyography signals in the second. These modalities were fused through decision-level integration within DF-Net, and the trained model consistently outperformed a traditional ML classifier (Random Forest), achieving superior recognition accuracy across multiple feature subsets and highlighting the effectiveness of deep multimodal fusion for procedural skill assessment. Even though manual percutaneous coronary intervention (PCI) was conducted as the manual task, the framework focused on guidewire manipulation and multimodal skill recognition relevant to vascular interventional robotics.

### 3.3 Natural language processing

NLP emerged in the late 1960s when Weizenbaum (66) developed the first NLP-based computer program and chatbot, ELIZA, which imitated a psychotherapist (67). This pioneering work inspired decades of subsequent research and continues to influence the field through the rapid development of LLMs. NLP focuses on the computational analysis and interpretation of human language by capturing its contextual and semantic meaning (68), thereby enabling its application to healthcare documentation and clinical records (Figure 3). The NLP studies discussed in this subsection are summarized in Table 3.

**Table 3:** AI in endovascular minimally invasive surgery: natural language processing.

Study	Application	Sample data	Reported results	Validation
Gaviria-Valencia et al. (69)	Real-time AAA diagnosis from radiology reports using rule-based NLP	Mayo Clinic: 360 radiology reports	PPV: 0.98; Acc: 0.97; F1: 0.97	Internal and Prospective
McLenon et al. (70)	AAA detection and maximal aortic diameter extraction from radiology reports using NLP	KPNC registry: 230,660 imaging reports from 32,778 patients	(AAA): PPV: 0.95; Sens: 0.885; F1: 0.916. (Maximal diameter): PPV: 0.888; Sens: 0.882	Internal
Weissler et al. (71)	Peripheral artery disease (PAD) diagnosis from EHR using label-embedding attentive model	Duke University Health System: 6,861 patients	AUROC: 0.89	Internal
Killeen et al. (72)	Voice-controlled LLM interface for intraoperative X-ray assistance	212 prompts by attending physician	Satisfactory actions: 0.97	Phantom

AAA, abdominal aortic aneurysm; Acc, accuracy; AI, artificial intelligence; AUROC, area under the receiver operating characteristic curve; EHR, electronic health record; LLM, large language model; NLP, natural language processing; PAD, peripheral artery disease; PPV, positive predictive value.

A study by Gaviria-Valencia et al. (69) developed a rule-based NLP algorithm capable of diagnosing AAA using patient radiology reports. The automated extraction of clinically relevant information enabled near real-time AAA identification, improving radiologist productivity by reducing reporting workload. The algorithm demonstrated strong diagnostic performance, achieving high accuracy, sensitivity, and specificity, and was therefore established as a reliable decision-support tool for clinical practice. Similarly, McLenon et al. (70) evaluated the effectiveness of a commercially available NLP system in detecting the presence of AAA and determining maximal aneurysm diameter from large-scale radiology reports. By extracting clinically meaningful textual features, the algorithm achieved high accuracy in identifying both AAA presence and maximal diameter, highlighting the potential of NLP for enhanced AAA surveillance, operative decision-making, and longitudinal patient follow-up.

Beyond AAA detection, NLP models were also applied in the diagnosis of PAD. A study by Weissler et al. (71) introduced PAD-ML, an NLP algorithm built upon a label-embedding attentive model, facilitating the diagnosis of PAD by identifying clinical context within electronic health records and clinical notes. The predictive performance of PAD-ML outperformed the traditional structured data-based approach with higher precision and specificity, displaying the advantages of leveraging NLP models for disease diagnosis and real-time decision-making.

Furthermore, several studies have proposed the integration of NLP algorithms and LLMs as virtual assistants to support healthcare professionals across a range of clinical tasks (68, 73). One particularly promising application is the development of intraoperative virtual assistants. Killeen et al. (72) introduced a voice-controlled, LLM-integrated interface for a robotic C-arm X-ray imaging system, enabling surgeons to access high-level system functions. These capabilities include intraoperative image analysis and automated landmark detection, contributing to reduced radiation exposure and shorter anesthesia duration.

Despite the demonstrated potential, NLP has primarily been used as a preoperative planning tool for patient record analysis and vascular diagnosis. The use of NLP in endovascular MIS remains comparatively limited relative to ML, DL, and robotics in the literature analyzed in this review. However, we anticipate that greater emphasis will be placed on the integration of LLM-driven virtual assistants within surgical robotics, particularly for technically demanding procedures such as endovascular interventions.

### 3.4 Robotics and reinforcement learning

In the broader history of surgery and robotics, two milestones laid the early groundwork for the development of MIS. The first was the introduction of the PUMA 200 robot in 1985, which was used to perform a stereotactic brain biopsy and represented one of the earliest instances of robotic assistance in surgery. The second was the advancement of endoscopy in the 1960s by Hopkins, which revolutionized visualization in minimally invasive procedures. Although neither innovation was specific to endovascular surgery, they respectively established the foundations of surgical robotics and MIS, contributing substantially to their subsequent evolution. Within endovascular MIS, two principal categories of robotic systems have emerged: fixed and consolidated robot-assisted MIS platforms, and continuum robots featuring snake-like, deformable structures (74). These systems support a wide range of procedures, including balloon stenting, thrombectomy, catheter ablation, and targeted drug delivery (75). Over time, the field has undergone continuous innovation, progressing from the first endovascular robotic platform, CorPath 200, introduced in 2010 (74), to the more recent R-One+ system launched in 2023 (76). These platforms incorporate one or more core technologies, such as catheter-based instrument delivery, haptic feedback, surgical navigation, and virtual surgical systems (75). As an inherently interdisciplinary domain, robotic systems increasingly integrate ML, DL, NLP, and RL for various applications, including surgical tool segmentation and autonomous navigation (Figure 4). The robotics and RL studies discussed in this subsection are summarized in Table 4.

**Figure 4:**
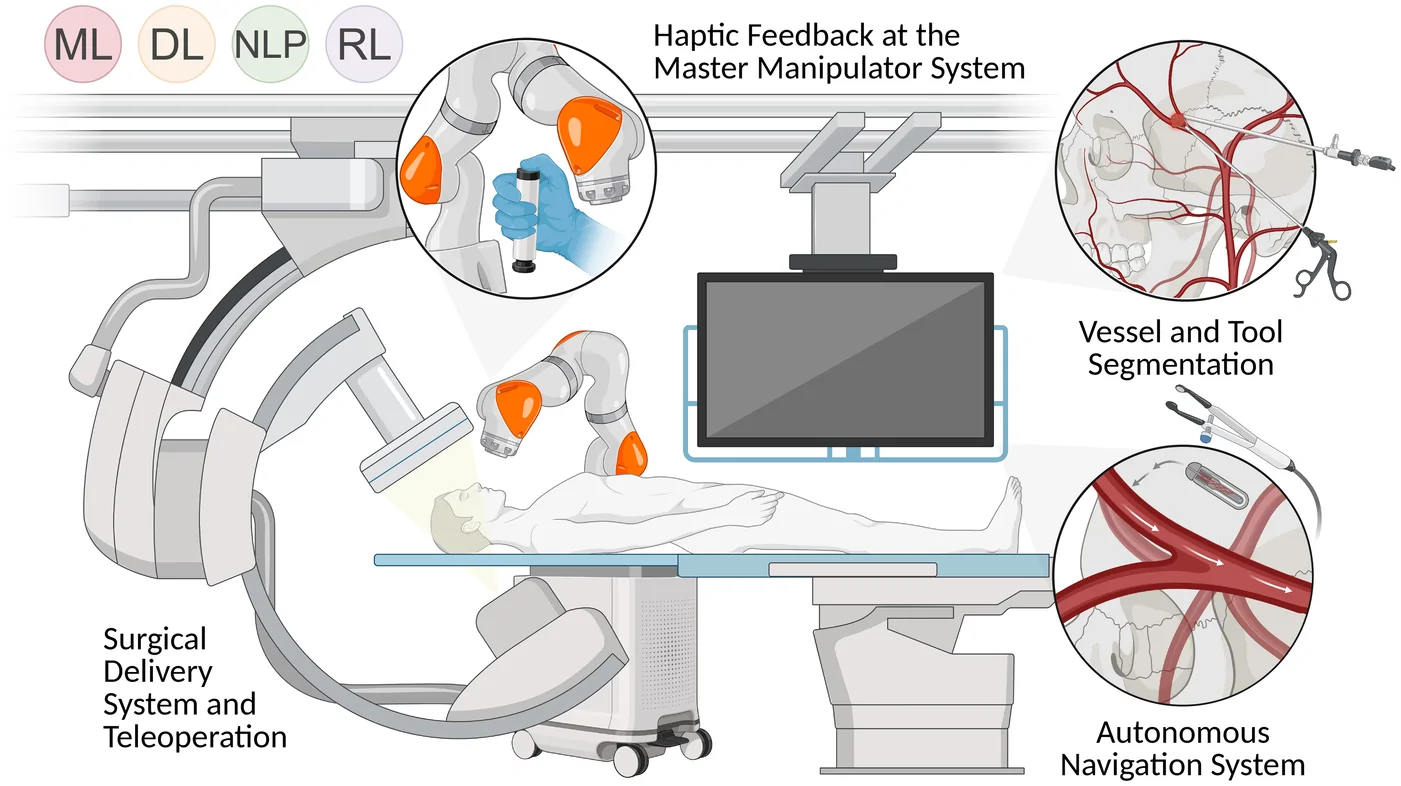
Core technologies used in robotic endovascular MIS, including the surgical delivery system, haptic feedback, surgical navigation, and navigation system in a clinical setting. Created in BioRender. Jiang, C. (2026) https://biorender.com/qln0zpj.

**Table 4:** AI in endovascular minimally invasive surgery: robotics.

Study	Application	Validation setting/sample	Reported results
Mahmud et al. (77)	Robot-assisted femoropopliteal angioplasty for PAD	Prospective human trial: 20 patients, 29 lesions	Success rate: 1.00 (technical), 1.00 (clinical)
Omisore et al. (78)	Guidewire segmentation using multilateral branched deep network	Animal + Phantom: 2,700 angiograms	Single-guidewire: mIoU: 0.90; AUROC: 0.94. Multi-guidewire: mIoU: 0.85; AUROC: 0.91
Zhang et al. (79)	Robotic catheter ablation with tactile force feedback	In vitro heart model: (multiple trials)	Axial: ±0.98; radial: ±1.80; bending: ±1.90; absolute: ±2.00
Chen et al. (80)	Portable autonomous robotic cannulation system with automated segmentation and real-time servoing	In vitro; in vivo	NIR: Dice: 0.80; MHD: 2.52 mm. US: Dice: 0.84; MHD: 0.54 mm
Wei et al. (81)	Telemanipulated vascular intervention system for robot-assisted percutaneous coronary and neurovascular interventions	In vitro; in vivo: 2 rabbits	Wire RMSE: 0.03–0.05 mm; catheter RMSE: 0.04–0.06 mm; rotational RMSE: $16.18^{\circ }$
Ligtenberg et al. (82)	Magnetic untethered robot for improved vascular access	Ex vivo porcine aorta	Maximum flow: 67 mL/min; biofilm reduction: >0.99
Song et al. (83)	Autonomous endovascular navigation with localization and mapping	Phantom: 25 trials	Success rate: 1.00
Yang et al. (84)	DQN-based autonomous guidewire navigation system with integrated U-Net segmentation and tip tracking algorithm	Cerebral neovascular model: 60,000 iterations	Success rate: 0.95
Wang and Zhang (85)	Deep RL-based autonomous guidewire navigation	Simulated human vascular model	Success rate: >0.94

AI, artificial intelligence; AUROC, area under the receiver operating characteristic curve; DQN, deep Q-network; MHD, modified Hausdorff distance; mIoU, mean intersection over union; NIR, near-infrared; PAD, peripheral artery disease; RL, reinforcement learning; RMSE, root mean square error; US, ultrasound.

These advances have been accompanied by rigorous testing to attain higher safety measures. Mahmud et al. (77) evaluated the feasibility of robot-assisted balloon angioplasty using the CorPath 200 system in the femoropopliteal artery of 20 patients with symptomatic PAD, treating a total of 29 lesions. Technical success was defined as successful robotic cannulation and balloon delivery, while clinical procedural success was defined as below 50% residual stenosis without unexpected conversion to manual operation or device-related adverse events. The study reported a 100% success rate for both technical and clinical outcomes, establishing the safety and feasibility of robot-assisted peripheral vascular interventions.

Advanced segmentation techniques are essential for accurate mapping and visualization during robotic endovascular procedures, complementing both teleoperation and navigation. Omisore et al. (78) introduced MLB-Net, a multilateral branched network designed for pixel-level guidewire segmentation in angiographic images. Using encoder–decoder feature extraction combined with depth-wise attention mechanisms, MLB-Net demonstrated robust real-time performance while addressing challenges involving background noise and low-level contrasts. Compared with existing segmentation models such as SegNet and U-Net, MLB-Net achieved faster inference and higher segmentation accuracy, enhancing tool visualization and tracking during perioperative endovascular interventions.

Haptic feedback systems further enhance robotic endovascular MIS by restoring tactile sensation during teleoperated procedures, thereby improving surgical precision and efficiency (86). For example, Zhang et al. (79) introduced a novel haptic-feedback-integrated ablation catheter system employing master–slave manipulators. The master manipulator enables surgeons to control the guidewire and catheter motion via a control lever, while magnetorheological fluids generate real-time tactile feedback and collision detection through magnetic field modulation. This design enhances situational awareness and reduces collision forces. The slave manipulator executes surgical actions using a non-fixed catheter, enabling improved precision and omnidirectional rotation. Experimental validation by the authors demonstrated an axial error below ±0.98 mm and a radial error within ±1.8 degrees, while tactile-feedback collision detection reduced maximum collision forces and task-completion time among novice operators in an in vitro evaluation. The authors further suggested that shorter task duration could reduce radiation exposure, supporting the potential of the system to improve procedural safety.

Despite rapid advances in robotic endovascular systems, most studies of autonomous delivery and navigation were conducted in preclinical environments. Similarly, the studies analyzed below were assessed in simulation, phantom, ex vivo, or animal models, which allow controlled assessment of technical performance but do not establish clinical safety or readiness.

Advances in robotic technology have driven extensive research into optimizing delivery systems and teleoperation in endovascular MIS. A notable breakthrough occurred in 2020 when Chen et al. (80) introduced a portable, autonomous, image-guided robotic system capable of performing vascular cannulation in deformable tissues. The system integrates a deep CNN–based bimodal vessel imaging framework to encode spatiotemporal information, enabling automated vessel segmentation, classification, and real-time servo control for continuous adjustment of both the robotic arm and needle trajectory. Through this real-time image-guided approach, the device autonomously performs blood sampling or fluid delivery into peripheral vessels. Evaluation using phantom and animal models demonstrated superior performance compared with trained human operators, with higher success rates and reduced procedure times.

In a related study, Wei et al. (81) introduced a telemanipulated vascular intervention (TVI) system designed for minimally invasive coronary and neurovascular procedures. The TVI platform integrates a joystick, a delivery device, and a graphical user interface within an ergonomic architecture, enabling effective teleoperated intravascular intervention. Performance evaluation using in vitro three-dimensional cardio-cerebrovascular models and in vivo rabbit models for renal artery angioplasty demonstrated precise navigation across multiple types of coronary stenosis and cerebral arteries. The system also successfully accessed an intracranial aneurysm, supporting the preclinical feasibility and adaptability.

Beyond tethered robotic systems, Ligtenberg et al. (82) investigated the feasibility of blood-compatible, untethered magnetic robots (UMRs) to address challenges related to limited control, vessel accessibility, and navigational precision. The UMRs were specifically designed to access anatomically challenging locations, with potential applications in treating diseases that cause vascular blockages and clot formation. Under magnetic actuation, the UMRs were evaluated using an ex vivo porcine aorta model, demonstrating successful navigation, teleoperation, and directional control. The reduced size enabled superior accessibility compared with conventional guidewires and catheters, offering improved flexibility and maneuverability. Moreover, the hemocompatible surface coating significantly reduced bacterial adhesion, further enhancing the technical feasibility of UMRs as potential vascular delivery platforms.

While optimizing delivery systems and teleoperation remains essential, effective navigation strategies are equally critical for robotic endovascular interventions. Song et al. (83) proposed VascularPilot3D, the first fully autonomous three-dimensional endovascular navigation system based on real-time imaging. Autonomous navigation was achieved through an integrated mapping, localization, and navigation workflow. Existing algorithms were adapted to incorporate three-dimensional topological constraints, improving localization accuracy. A novel tree-based data structure was also introduced to represent vascular anatomy, enabling efficient global path planning. VascularPilot3D achieved a 100% success rate across five navigation tasks and outperformed both an RL-based navigation system and an expert surgeon in phantom experiments, demonstrating strong technical performance.

Similarly, Yang et al. (84) proposed an autonomous guidewire navigation framework based on a deep Q-network (DQN) reinforcement learning algorithm. The system integrates DQN with a U-Net–based vascular segmentation and guidewire tip tracking module to enable autonomous motion control. To accelerate learning and improve navigation accuracy, the framework employs a discretized state space and a customized reward function combining sparse and immediate rewards. A novel shortest-path distance metric was also introduced to mitigate fluctuations associated with Euclidean distance and prevent entrapment in local optima, referring to suboptimal navigation states in which the agent ceases further exploration before reaching the target during navigation through tortuous vasculature. Following training, the system outperformed inexperienced human operators within the experimental environment, achieving higher success rates while reducing the number of required maneuvers.

Furthermore, Wang and Zhang (85) introduced a novel autonomous guidewire navigation system based on proximal policy optimization, a deep reinforcement learning algorithm. A UNet++ architecture combined with B-spline curve modeling was employed for real-time guidewire segmentation and accurate directional control. The autonomous guidewire was evaluated in two human-simulated vascular branching models, achieving success rates of 96% and 94%, while eliminating the need for environmental modeling, invasive sensors, and manual dexterity.

## 4 Translational pathway and deployment

Due to the large numbers of trainable parameters, frequently relying on non-transparent “black-box” architectures, multiple risk-mitigating approaches have been proposed by the healthcare authorities in order to ensure both patient safety and the trustworthiness of AI deployment. Engineering of commercialized AI-based products for further implementation in clinical settings follows a hybrid algorithm consisting of a centrally developed and validated core model, which is then locally integrated into each hospital’s data pipelines and workflows, with site-level validation and continuous performance monitoring under the institution’s specific population, practice patterns, and regulatory requirements (87).

Paired with early stakeholder engagement, multidisciplinary working groups define the intended use case and success metrics through formal articulation of the clinical problem and identification of workflow gaps that can be addressed using AI-integrated solutions (88, 89). However, successful translation depends on more than algorithmic performance, as clinical deployment is also shaped by organizational, workflow, and sociotechnical barriers (90, 91).

After establishing the clinical and operational requirements, standardized “big data” representations, including task-agnostic foundation models, are then pre-trained on an aggregated data corpus and interoperability standards to capture generalizable medical knowledge for high-performance processing (92).

A combination of systematic data selection, curated subset labeling, and multi-party computation establishes a shared knowledge ecosystem structured for pre-market regulatory assessment (93). This unified approach enables the definition of standardized performance benchmarks and harmonized quality criteria while preserving patient privacy by design. However, benchmarking and evaluation practices may introduce bias that inflates reported performance relative to real-world behavior (94).

After pre-market assessment, safety, effectiveness, and necessary modifications are subsequently evaluated by regulatory authorities such as the European Medicines Agency and the Food and Drug Administration (FDA), following consensus-based reporting frameworks for software as a medical device, including CONSORT-AI and SPIRIT-AI (95, 96). However, regulatory authorization does not necessarily establish clinical effectiveness. Of 521 FDA-authorized medical AI devices examined by Chouffani El Fassi et al. (97), 226 lacked publicly available clinical validation data, while only 17 of 130 FDA-approved device evaluations reported performance across demographic subgroups (98).

Following regulatory clearance, locally tailored model adaptation is required to address demographic- and context-specific variations, thereby identifying and correcting potential biases arising from shifts in disease prevalence, treatment protocols, or device utilization (99). Institution-specific data subsets inform model retraining and validation, often through pragmatic randomized controlled trials comparing clinician-only care with AI-augmented clinical workflows (96).

Facility-specific documentation and decision-support processes should be integrated at the institutional level, ensuring compatibility with existing record archiving systems and continuous monitoring infrastructures (100). This integration enables post-deployment institutional review to assess patient outcomes, model performance, and real-world effectiveness over extended periods (101). Beyond clinical benefit, robust health economic evaluation is essential for adoption decisions. Experiences from surgical robotics illustrate how fixed capital costs (e.g., systems priced between $1–2.5 million), substantial training requirements, and added per-procedure variable costs challenge cost-effectiveness and reimbursement alignment of each project (102).

Post-market surveillance forms a core component of the continuous improvement strategy. Institutional performance data are streamed back to a central repository, where output metrics, safety signals, and subgroup-specific outcomes are audited to rapidly detect performance drift or degradation. Robust regulatory governance, reinforced by reporting benchmarks and auditable decision trails, establishes the trust framework necessary for sustained AI-assisted clinical care. Nevertheless, legal and liability considerations remain significant barriers to implementation. In the absence of comprehensive legal governance addressing medical AI–related harm, prevailing tort principles continue to prioritize the established standard of care. As a result, clinicians are generally protected when adhering to accepted clinical practice but may face increased liability when deviating from accepted standards and adverse outcomes occur. For this reason, current AI-enabled systems are predominantly positioned as confirmatory decision-support tools rather than justifications for non-standard clinical actions (103) (Figure 5).

**Figure 5:**
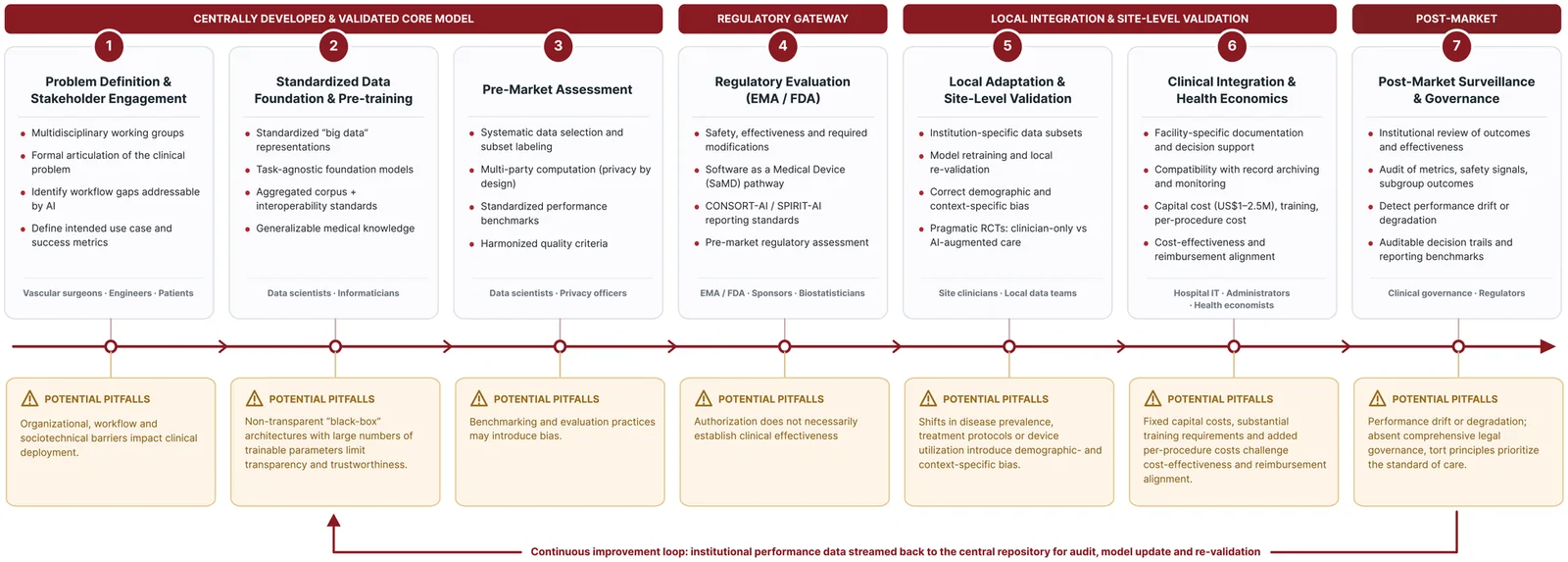
Translational pathway for AI-based solutions in vascular MIS, from problem definition to post-market surveillance.

## 5 Challenges and ethical considerations

With the growing demand for AI implementation in endovascular MIS, several concerns arise regarding the safe and controlled adoption of AI technologies in medical practice. As outlined in previous sections, AI applications in endovascular MIS demonstrate considerable potential. However, this potential is accompanied by inherent limitations, making it essential to address the associated technical and ethical challenges to ensure reliable and responsible integration into clinical workflows (Figure 6).

**Figure 6:**
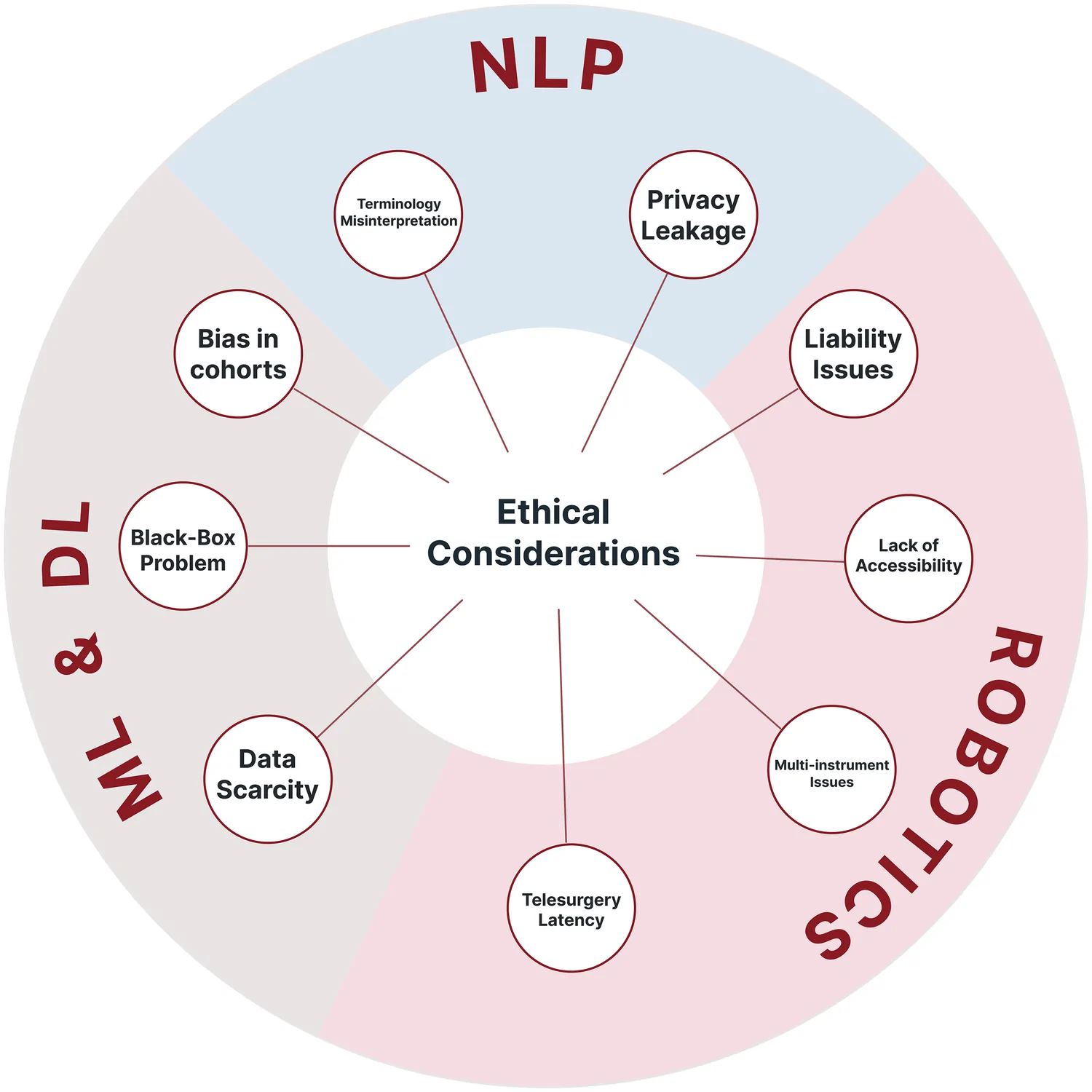
Ethical considerations associated with AI-assisted endovascular minimally invasive surgeries. Key technical, clinical, and regulatory challenges across AI modalities include the black-box nature of ML/DL models, cohort bias, data scarcity, terminology misinterpretation, and privacy leakage in NLP systems, limited accessibility, liability ambiguity, telesurgery latency, and multi-instrument incompatibilities. These considerations highlight the complexities of safely integrating AI technologies into vascular MIS and emphasize the need for transparent, equitable, and reliable deployment.

With respect to ML and DL, several co-occurring limitations have been identified. First, numerous studies have highlighted data-related challenges, whereby large volumes of homogeneous, high-quality data are required to achieve optimal model performance and improved generalizability (104). Second, cohort bias, particularly the exclusion or underrepresentation of minority populations, remains a major concern, as biased training data can compromise model predictions and reduce performance across diverse patient groups (27, 105). Beyond cohort composition, ML systems have been observed to reproduce racial disparities even when race is not explicitly included as a model input, particularly when “ground truth” labels rely on convenient proxies. For example, a widely deployed commercial risk-scoring algorithm used to allocate high-risk care management resources was shown to exhibit substantial racial bias, with Black patients found to be considerably sicker than White patients at comparable risk scores (106). This disparity arose because the algorithm was trained to predict future healthcare costs rather than illness severity; because Black patients incurred lower healthcare costs than White patients conditional on an equivalent burden of chronic illness, accurate cost prediction translated into systematically biased estimates of health need.

Third, although the decision-making capacity of ML algorithms offers substantial potential, the fact that model reasoning often exceeds human interpretability raises serious concerns regarding reliability and transparency (105). This issue, commonly referred to as the “black-box” problem (107), has prompted significant debate in clinical AI deployment. In response to these challenges and the frequent incompleteness of ML and DL model reporting, the TRIPOD+AI reporting guideline was introduced as an extension of TRIPOD 2015. This framework provides a harmonized checklist for transparent reporting of prediction models developed or evaluated using regression or machine-learning approaches, thereby facilitating critical appraisal, reproducibility, and safer clinical translation (108). Consequently, post-hoc methods have become widely used to interpret black-box models. However, Rudin (109) argues that post-hoc explanations are inherently unreliable and instead advocates for the use of inherently interpretable ML models. Such models enable direct insight into the internal decision-making process, distinguishing them from post-hoc approaches. Although inherently interpretable models are often domain-specific, several have been developed for healthcare applications, including optimal sparse scoring systems, additive models, uncertainty-constrained recovery curve models, and prototype-based convolutional neural networks (109). Nevertheless, as SHAP is a post-hoc attribution technique rather than an inherently interpretable model, these studies do not constitute true examples of inherently explainable ML. Finally, the autonomous decision-making capabilities of ML models introduce ambiguity regarding clinical liability and responsibility towards patients, underscoring the need for updated legal and ethical guidelines (104).

Beyond model interpretability, limited external validation represents another important challenge towards the clinical translation of ML- and DL-based systems. Cross-validation or internal testing performance may not be sustained when models are applied across different institutions, patient populations, imaging systems, treatment practices, or healthcare environments. A recent meta-analysis by Ravindhran et al. (110), focusing on predictive ML models in PAD patients, reported that only 3 of 13 studies conducted external validation. Moreover, a broader systematic review by Li et al. (27) reported that only 9 of 212 ML studies in vascular surgery have conducted external validation, with 94.8% of these studies displaying high overall risk of bias and 41.4% demonstrating low reporting standards. This limitation is particularly important for models developed using small or single-center datasets, even though models derived from large multicenter databases may also remain vulnerable to source-specific biases. Hence, independent external validation is necessary for model deployment, followed by prospective evaluation in real-world clinical settings to assess clinical utility and robustness.

In contrast to conventional ML and DL approaches, NLP models, particularly LLMs, present a distinct set of ethical challenges in medical applications. These challenges include limitations in medical terminology comprehension and concerns surrounding patient privacy. From a technical perspective, LLMs rely on word embeddings (111) and self-attention mechanisms (112), enabling them to generate highly fluent and human-like text (113). Although these techniques underpin strong linguistic performance, they remain fundamentally dependent on statistical patterns and internal representational states (114), leading to approximations of meaning rather than genuine semantic understanding as observed in human cognition (113). As a result, NLP pipelines may struggle to accurately interpret complex medical terminology, particularly in the context of acronym disambiguation (115), thereby impairing downstream performance.

Lexical ambiguity, whereby identical abbreviations represent multiple context-dependent biomedical concepts, poses a significant obstacle for clinical NLP systems and directly affects concept-mapping pipelines such as MetaMap (116, 117). Incorrect acronym expansion can lead to misalignment with inappropriate Unified Medical Language System (UMLS) concepts, propagating errors throughout subsequent information extraction and clinical decision-support processes. This degradation adversely impacts the reliability of automated chart review, clinical coding, and decision-support outputs. Large biomedical terminologies, including the UMLS Metathesaurus, contain numerous ambiguous entries that necessitate robust disambiguation strategies. Benchmark datasets and annotated corpora, therefore, play a critical role in leveraging local clinical context to train and validate acronym-resolution models, enabling more reliable concept normalization in clinical settings (118). In addition, privacy remains a central ethical concern, as NLP systems in endovascular MIS are typically applied to electronic health records containing highly sensitive patient data.

Robotics and reinforcement learning introduce further challenges and ethical considerations within endovascular MIS. These challenges include regulatory uncertainty and liability concerns arising from the absence of a comprehensive legal framework (119, 120). The significance of this issue arises from reports of manufacturers and healthcare institutions overstating the benefits of robotic surgery despite limited empirical evidence and documented adverse outcomes, thereby violating the principles of informed consent (120, 121).

Cautionary implementation failures in adjacent clinical AI deployments illustrate how public and media expectations can outpace real-world performance and governance. For instance, the IBM Watson-based Oncology Expert Advisor project at MD Anderson Cancer Center was suspended after a substantial expenditure of US$62 million, due to its occasionally unsafe or low-concordance recommendations (e.g., a reported 49% concordance in one colon cancer cohort) and a software bug that recommended surveillance instead of aggressive treatment for certain metastatic cases, with limited evidence demonstrating improved patient outcomes (122). With rapidly advancing technology, surgical robots are getting more autonomous, which could potentially amplify the current liability issues to a greater extent, necessitating an update to the existing legal framework.

Additional ethical concerns include economic inequality and insufficient training. The high costs associated with the manufacture and deployment of surgical robotic systems limit affordability and restrict access to robotic procedures (123). While current robotic platforms perform well in routine vascular procedures, their effectiveness decreases in complex MIS requiring simultaneous manipulation of multiple instruments, highlighting the need for advanced multi-instrument robotic systems (124).

Finally, greater emphasis should be placed on remote telesurgical applications (125), which offer substantial clinical and logistical benefits. Despite being technically feasible, widespread adoption remains limited by requirements for high-resolution visualization, precise haptic feedback, and ultra-low latency communication, posing significant barriers to practical implementation (124).

## 6 Future perspectives

Building on these limitations, future progress in AI-assisted endovascular MIS should prioritize independent temporal and external validation rather than algorithmic performance alone, expanding beyond common performance metrics such as AUROC and accuracy. Future studies can adopt several reporting and methodological assessment frameworks such as TRIPOD+AI (108) and PROBAST + AI (126) to facilitate more transparent and reproducible validation.

After external validation, prospective silent-mode deployment acts as an important step before AI outputs are incorporated into clinical decision-making for healthcare, enabling responsible clinical deployment (127). According to Tikhomirov et al. (128), the term “silent” refers to the prospective testing of the AI model in the intended clinical environment while the generated outputs are hidden from the clinicians, enabling the evaluation of both potential utility and financial sustainability. The effects of silent evaluation can be observed in a study by Kwong et al. (129), where the authors reported a decline from 0.90 to 0.50 on model AUROC during model development and initial silent evaluation, respectively. However, the authors fixed the observed issues after the initial silent trial, resulting in a model AUROC of 0.85 and 0.91 on two silent datasets and demonstrating how deployment-related performance issues may remain undetected during conventional retrospective testing. Future studies with promising prospective performance can proceed to initial human-in-the-loop evaluations such as DECIDE-AI (130), followed by other clinical trials to assess the effects of clinical decisions, workflow, and safety.

Alongside prospective clinical studies, future studies should incorporate health-economic and budget-impact analyses to support sustainable clinical implementation. A recent study by Godoy Junior et al. (131) assessed health-economic evaluations of 117 medical AI studies, revealing that only 28% incorporated implementation costs. Only 57% of the studies included operational costs, hindering transparency regarding cost-effectiveness. Hence, before deployment, future endovascular studies should assess whether AI provides clinical or operational value compared to standard healthcare. Specifically, robotic surgical systems should be more transparent regarding health economic evaluations since high acquisition, maintenance, and operating costs may limit widespread clinical adoption (132).

Moreover, these economic barriers may also contribute to inequitable accessibility, as costly AI or advanced MIS infrastructure may be concentrated within well-resourced centers, restricting availability for economically disadvantaged populations and safety-net hospitals (133, 134). Beyond accessibility, equitable implementation also requires AI models to perform consistently across demographically diverse patient populations. Accordingly, future studies should include representative cohorts, evaluate model generalizability across demographic and socioeconomic groups, and adhere to strict reporting standards to mitigate bias in vascular care (135).

Lastly, as discussed in previous sections, liability remains a major concern with increasing robotic autonomy, the growing role of ML-based decision-making models, and the absence of comprehensive legal frameworks (104, 119, 120). Hence, future studies should focus on establishing clear accountability among clinicians, healthcare institutions, and manufacturers, while documenting AI-assisted decisions.

## 7 Limitations

This narrative review has several limitations. First, the review was not conducted as a systematic review and therefore did not employ a predefined systematic search and screening protocol. The literature search relied primarily on Google Scholar. In addition, publications were considered inaccessible when the full text could not be obtained through the authors’ institutional subscriptions or publicly available sources. Corresponding authors were not contacted for additional access. Moreover, abstract-only publications without an accessible full-text article, preprints, unpublished manuscripts, and non-peer-reviewed material were excluded. Although this approach prioritizes peer-reviewed evidence, it may restrict the inclusion of the most recent developments in a rapidly evolving field such as AI and robotics. Furthermore, some of the included studies varied in sample size, study design, clinical application, datasets, validation methods, and reported performance metrics, compromising direct comparisons between AI approaches.

The conclusions of this review are also constrained by limitations within the available evidence base. As discussed throughout this review, many AI models were evaluated primarily through internal validation, while several robotic and autonomous systems were tested mainly in simulation, phantom, ex vivo, or animal settings, limiting conclusions regarding reproducibility, generalizability, and clinical readiness.

## 8 Discussion and conclusion

Compared with previous reviews, this study provides an updated analysis, focusing on AI applications in endovascular MIS. Earlier reviews have investigated individual AI techniques, including ML and broader AI applications in vascular surgery (27, 136), NLP applications (68), robot-assisted endovascular applications (124), and autonomous robotic navigation (74, 137). Conversely, this review integrates ML, DL, NLP, and robotics with RL into a unified endovascular MIS framework and includes recent developments through September 2026. Moreover, this review examines the translational pathways of AI modalities towards clinical implementation and discusses important factors such as validation, silent-mode deployment, cost, equity, and liability for safer deployment.

In summary, the evidence from the reviewed studies demonstrates distinct roles for each AI method in vascular MIS across the perioperative stages. ML-based models display potential for patient risk stratification and outcome prediction, supporting patient selection, preoperative decision-making, and postoperative care. DL-based models support image-based planning, anatomical segmentation, and intraoperative visualization. NLP-based approaches primarily assist with the extraction and interpretation of clinical information from electronic health records. Robotic and reinforcement-learning systems may further support navigation, surgical delivery, and procedural assistance. However, the strength of the evidence remains inconsistent, as many predictive models lack independent external and prospective validation. Moreover, several autonomous robotic systems have primarily been evaluated in simulation, phantom, ex vivo, or animal settings. These limitations restrict conclusions regarding the generalizability and clinical effectiveness of certain AI-based approaches.

Hence, future research should move beyond algorithmic performance and establish measurable clinical and economic benefits in vascular MIS. Specifically, prospective multicenter studies should be prioritized to assess improvements associated with AI-based approaches in patient selection, preoperative planning, procedural safety, and patient outcomes across the perioperative pathway. Furthermore, implementation, maintenance, training, and workflow costs should be evaluated to determine whether AI-assisted vascular MIS provides meaningful benefits compared with standard care. External validation, prospective deployment, standardized reporting, health-economic assessment, and evaluation across diverse patient populations will be important for translating technical performance into safe, effective, and sustainable clinical practice.

## Glossary

**Table:**

AAA	abdominal aortic aneurysm
ACS NSQIP	American College of Surgeons National Surgical Quality Improvement Program
Acc	accuracy
AI	artificial intelligence
ANN	artificial neural network
AUC	area under the curve
AUROC	area under the receiver operating characteristic curve
CHASE DB1	Child Heart and Health Study in England Database 1
CNN	convolutional neural network
CONSORT-AI	Consolidated Standards of Reporting Trials-Artificial Intelligence
CT	computed tomography
CV	computer vision
DBCM	dual-branch convolutional module
DECIDE-AI	Developmental and Exploratory Clinical Investigations of DEcision support systems driven by Artificial Intelligence
DF-Net	data fusion network
DL	deep learning
DQN	deep Q-network
DRIVE	Digital Retinal Images for Vessel Extraction
DSA	digital subtraction angiography
DTA	decision tree analysis
EHR	electronic health record
EMA	European Medicines Agency
EMR	electronic medical record
EVAR	endovascular aneurysm repair
F1	F1 score
FDA	Food and Drug Administration
FR-UNet	full-resolution network
GAN	generative adversarial network
GenePAD	Genetic Determinants of Peripheral Arterial Disease
IoU	intersection over union
IT	information technology
KPNC	Kaiser Permanente Northern California
LLM	large language model

mAP	mean average precision
MCE	maximum calibration error
MHD	modified Hausdorff distance
MIS	minimally invasive surgery
ML	machine learning
MLB-Net	multilateral branched network
mIoU	mean intersection over union
N/A	not applicable
NIR	near-infrared
NLP	natural language processing
NR	not reported
PAD	peripheral artery disease
PCI	percutaneous coronary intervention
PERSIST	Posterior Circulation Ischemic Stroke Registry
PPV	positive predictive value
PROBAST + AI	Prediction model Risk Of Bias ASsessment Tool + Artificial Intelligence
RCT	randomized controlled trial
ReLU	rectified linear unit
RL	reinforcement learning
RMSE	root mean square error
SAH	subarachnoid hemorrhage
SaMD	software as a medical device
Sens	sensitivity
SHAP	SHapley Additive exPlanations
Spec	specificity
SPIRIT-AI	Standard Protocol Items: Recommendations for Interventional Trials-Artificial Intelligence
3D	three-dimensional
TRIPOD + AI	Transparent Reporting of a multivariable prediction model for Individual Prognosis Or Diagnosis + Artificial Intelligence
TVI	telemanipulated vascular intervention
UMLS	Unified Medical Language System
UMR	untethered magnetic robot
US	ultrasound
VD	vascular difficulty
VQI	Vascular Quality Initiative
XGBoost	eXtreme Gradient Boosting
